# Tumor Location Relative to the Spleen Is a Prognostic Factor in Lymphoma Patients: A Demonstration from the REMARC Trial

**DOI:** 10.2967/jnumed.123.266322

**Published:** 2024-02

**Authors:** Kibrom B. Girum, Anne-Ségolène Cottereau, Laetitia Vercellino, Louis Rebaud, Jérôme Clerc, Olivier Casasnovas, Franck Morschhauser, Catherine Thieblemont, Irène Buvat

**Affiliations:** 1 LITO Laboratory, U1288 Inserm, Institut Curie, University Paris-Saclay, Orsay, France;; 2 Department of Nuclear Medicine, Cochin Hospital, AP-HP, Paris Descartes University, Paris, France;; 3 Department of Nuclear Medicine, Saint Louis Hospital, AP-HP, Paris, France;; 4 Research and Clinical Collaborations, Siemens Medical Solutions USA, Knoxville, Tennessee;; 5 Department of Hematology, University Hospital of Dijon, Dijon, France;; 6 Research Group on Injectable Forms and Associated Technologies, Department of Hematology, Claude Huriez Hospital, University Lille, Lille, France; and; 7 Department of Hematology, Saint Louis Hospital, AP-HP, Paris, France

**Keywords:** tumor location, FDG, CT, DLBCL, artificial intelligence

## Abstract

Baseline [^18^F]FDG PET/CT radiomic features can improve the survival prediction in patients with diffuse large B-cell lymphoma (DLBCL). The purpose of this study was to investigate whether characterizing tumor locations relative to the spleen location in baseline [^18^F]FDG PET/CT images predicts survival in patients with DLBCL and improves the predictive value of total metabolic tumor volume (TMTV) and age-adjusted international prognostic index (IPI). **Methods:** This retrospective study included 301 DLBCL patients from the REMARC (NCT01122472) cohort. Physicians delineated the tumor regions, whereas the spleen was automatically segmented using an open-access artificial intelligence algorithm. We systematically measured the distance between the centroid of the spleen and all other lesions, defining the SD of these distances as the lesion spread (SpreadSpleen). We calculated the maximum distance between the spleen and another lesion (Dspleen) for each patient and normalized it with the body surface area, resulting in standardized Dspleen (sDspleen). The predictive value of each PET/CT feature for progression-free survival (PFS) and overall survival (OS) was evaluated through univariate and multivariate time-dependent Cox models and Kaplan–Meier analysis. **Results:** In total, 282 patients (mean age, 68.33 ± 5.41 y; 164 men) were evaluated. The artificial intelligence algorithm successfully segmented the spleen in 96% of the patients. SpreadSpleen, Dspleen, and sDspleen were correlated neither with TMTV (Pearson ρ < 0.23) nor with IPI (Pearson ρ < 0.15). When median values were used as the cutoff, SpreadSpleen, Dspleen, and sDspleen all significantly classified patients into 2 risk groups for PFS and OS (*P* < 0.001). They complemented TMTV and IPI to classify the patients into 3 risk groups for PFS and OS (*P* < 0.001). Integrating SpreadSpleen, Dspleen, or sDspleen into a Cox model on the basis of TMTV, IPI, and TMTV combined with IPI significantly improved the concordance index for PFS and OS (*P* < 0.05). **Conclusion:** Baseline PET/CT features that characterize tumor spread and dissemination relative to the spleen strongly predicted survival in patients with DLBCL. Integrating these features with TMTV and IPI further improved survival prediction.

## Visual Abstract

**Figure:**
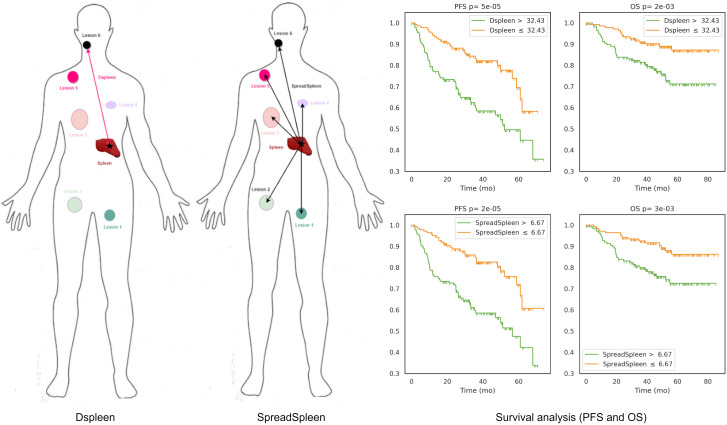

Whole-body [^18^F]FDG PET/CT is a standard of care for staging and assessing responses of patients with diffuse large B-cell lymphoma (DLBCL). The CT images are often preferred to view the anatomic structures, and the [^18^F]FDG PET images are used to capture the molecular activities of the tumor. Despite the widespread use of the age-adjusted international prognostic index (IPI) in DLBCL, recent literature suggests that image-based biomarkers could also be used for this purpose (1). Baseline [^18^F]FDG PET–based features that characterize the tumor burden, such as the total metabolic tumor volume (TMTV), have been shown to predict survival in DLBCL patients (1–4). Recently introduced tumor dissemination features, such as the distance between the 2 farthest lesions (Dmax) and the maximum distance between the largest lesion and another lesion (Dbulk), have shown promising results for predicting survival (5–9). Their simplicity, intuitive interpretation, and value in predicting the outcome inspired this study.

Given that the spleen plays a particular role in the lymphatic system and particularly in DLBCL (10), we assumed that the spleen could serve as a reference organ to model the disease distribution and dissemination over the whole body.

The purpose of this study was to investigate whether tumor distribution and dissemination measured relative to the spleen from baseline [^18^F]FDG PET/CT images had prognostic values independent of that of TMTV and IPI and improved survival prediction in DLBCL patients. The individual prognostic values of these new image-based features were evaluated in terms of progression-free survival (PFS) and overall survival (OS). We also assessed the added prognostic value of these biomarkers when they are combined with TMTV and age-adjusted IPI.

## MATERIALS AND METHODS

### Patients

**FIGURE 1.**
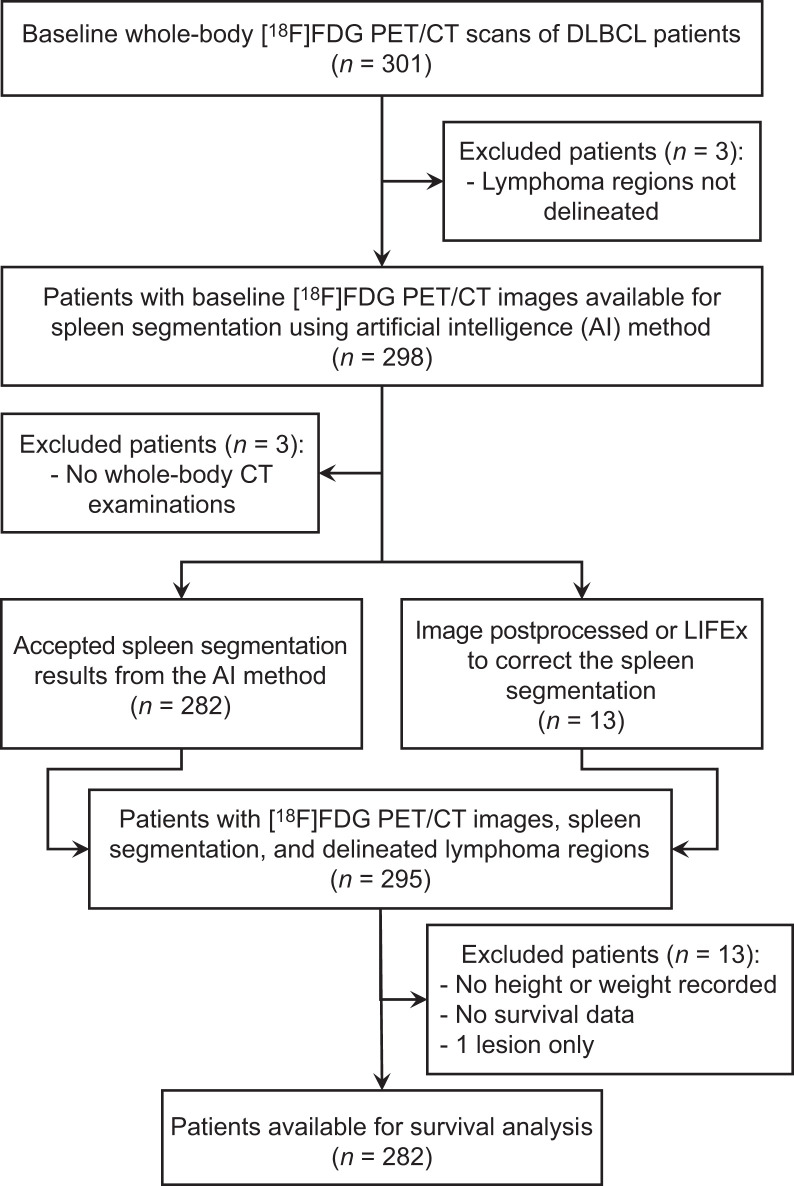
Study flowchart.

A retrospective analysis of 301 DLBCL patients with a baseline [^18^F]FDG PET/CT scan from the REMARC trial (NCT01122472) was conducted. The REMARC trial study started including patients in 2010 and was a double-blind, international, multicenter, randomized phase II study. Patients included in this study were 60–80 y old and had an Ann Arbor stage of I–IV and age-adjusted IPI of at least 1 at diagnosis, with histologically proven CD20-positive DLBCL according to the 2008 World Health Organization criteria (11). Detailed characteristics of the patients were reported elsewhere (2). The survival outcomes (PFS and OS) were recorded as defined by the revised National Cancer Institute criteria (12). Figure 1 summarizes the flow diagram of the data. Patient data were anonymized before any analysis. All patients gave written informed consent, and institutional review board approval, including ancillary studies, was obtained. The demographics and staging of the DLBCL patients used for the survival analysis are summarized in Table 1.

**TABLE 1.** Population Characteristics (*n* = 282)

Parameter	Number
Men	164 (58.2%)
Women	118 (41.8%)
Median age (y)	68.33 (64.0–73.0)
Median weight (kg)	72 (63.0–83.0)
Median height (cm)	168 (160.0–175.0)
Ann Arbor stage	
<I	1 (0.4%)
≥II	281 (99.6%)
Performance status	
0	114 (40.4%)
1	120 (42.6%)
2	41 (14.5%)
3	2 (0.7%)
4	2 (0.7%)
Missing	3 (1.1%)

Qualitative data are number and percentage; continuous data are median and interquartile ranges (quartile 1 to quartile 3).

### Image Analysis and Feature Extraction

Two experienced nuclear medicine physicians (with 7 and 10 y of experience) delineated the lesion regions semiautomatically from the baseline 3-dimensional PET/CT images. The exact delineation procedure has been previously described (2). All lesion segmentations were visually verified to include pathologic lesions and to exclude physiologic uptake. The total volume of the lesions for each patient was then calculated as TMTV. Two recently introduced lesion dissemination features, Dmax (6) and Dbulk (7), were calculated from each lesion’s centroid.

#### Automatic Spleen Segmentation on CT Images

**FIGURE 2.**
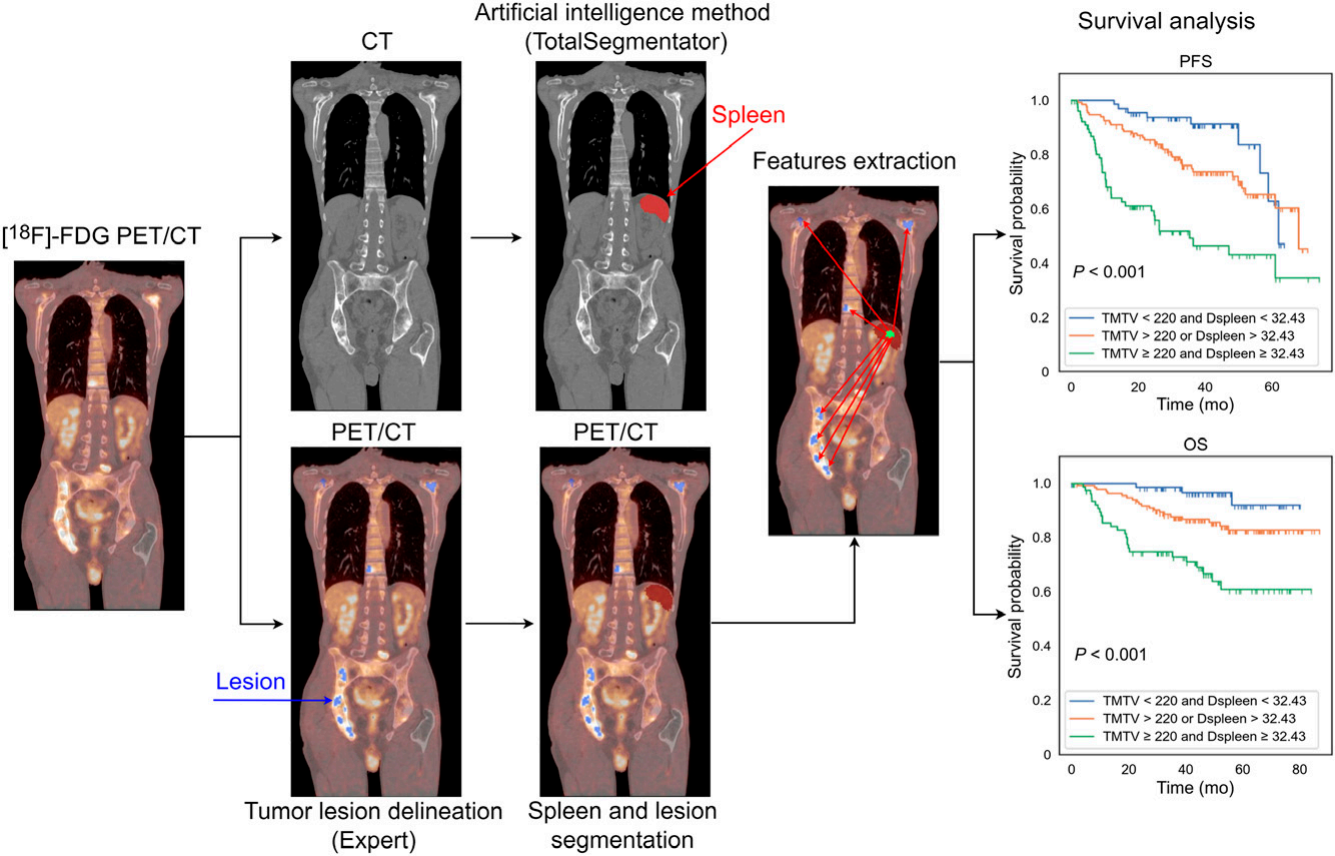
Overview of image-processing pipeline to calculate tumor locations relative to spleen from [^18^F]FDG PET/CT images. Deep learning–based whole-body segmentation was used to segment spleen from CT images. From overlapped tumor and spleen segmentations, we extracted whole-body radiomic features, including TMTV and Dspleen. Extracted radiomic features were used to predict OS and PFS.

An artificial intelligence (AI) method called TotalSegmentator was used to segment the spleen from 3-dimensional CT images (13). This method was initially developed to segment 104 anatomic structures from CT images, but in our work, we focused on spleen segmentation only. A postprocessing method was developed to check the quality of the spleen segmentation and correct it when needed. The postprocessing method included 2 criteria to trigger a warning, ensuring that the spleen segmentation had a single connected component and was not outside the whole-body field of view. If the spleen included more than 1 connected component, the largest one was automatically selected as the spleen. After postprocessing, all segmentations were visually verified and manually adjusted using the LIFEx software whenever needed (14). As the CT and PET images were aligned from the PET/CT acquisition, for each patient, the spleen segmentation result obtained from the AI was superimposed onto the lesion segmentation obtained from the experts. Figure 2 illustrates the proposed image-processing pipeline.

#### PET/CT Feature Extraction

The superimposed spleen and lesion 3-dimensional regions were used to calculate the tumor location relative to the spleen. From the 3-dimensional coordinates of the spleen region, the centroid was automatically calculated and defined the spleen location. For each patient, the distance between the centroid of the spleen, $A=\left(x_{a},y_{a},z_{a}\right)$, and all other detected lesion centroids $(B_{i}=x_{bi},y_{bi},z_{bi}$, centroid of the *i*th lesion) was measured using the Euclidean distance formula $\sqrt{{\left(x_{bi}-x_{a}\right)}^{2}+{\left(y_{bi}-y_{a}\right)}^{2}+{\left(z_{bi}-z_{a}\right)}^{2},}\;i=1,2,3\ldots ,N$ for *N* detected lesions. The SD of the distance between the spleen centroid and all lesions was calculated for each patient as $\text{σ}=\sqrt{\frac{\sum_{i=1}^{N}{\left(\text{ABi}-\text{μ}\right)}^{2}}{N}}$ and was referred to as lesion spread relative to the spleen (SpreadSpleen), where $\text{μ}=\sum_{i=1}^{N}(AB_{i})/N$. The distance between the spleen and the farthest lesion from the spleen (Dspleen) was deduced for each patient and normalized by the patient’s body surface area, given by $\text{body surface}\;\text{area}=\frac{\sqrt{\text{weight}\;(\text{kg})\times \text{height}\;(\text{cm})}}{60},$ and named hereafter the standardized distance of the farthest lesion from the spleen (sDspleen) (15). Supplemental Figure 1 illustrates the definitions of the new features relative to the spleen (supplemental materials are available at http://jnm.snmjournals.org). The image-processing pipeline, including the AI-based spleen segmentation, feature extraction, and survival analysis, is publicly available at https://github.com/KibromBerihu/TumorLocationProfiler.

The exact same analysis was performed using the liver as a reference organ, as described in the supplemental materials.

### Statistical Analysis

The predictive power of the calculated features was evaluated in univariate and multivariate analyses. First, we evaluated if each of the calculated biomarkers was predictive of the PFS and OS using Kaplan–Meier survival analysis and a time-dependent area under the receiver operating characteristics curve (AUC), and the results obtained for the different features were compared. Correlations between the biomarkers were calculated using the Pearson correlation coefficient (ρ). Second, we evaluated the added values of the predictive biomarkers combined with known predictive biomarkers, including the TMTV and the IPI. A multivariate Cox regression analysis with a bootstrap resampling at the patient level was used to associate CIs to the Cox model hazard ratio and the concordance index. The empiric 95% CI was reported with a bootstrap of 5,000 random samplings with replacement. Cox proportional hazard models were used to analyze univariate and multivariate results. *P* values less than 0.05 were interpreted as statistically significant.

## RESULTS

Among the 301 patients from the REMARC cohort, 282 patients (mean age, 68.33 ± 5.41 y; 164 men) were included for the biomarker and survival analysis. [^18^F]FDG PET/CT quality control and other criteria described in Figure 1 excluded 19 patients. The characteristics of the 282 patients included for the survival analysis are shown in Table 1.

### Spleen Segmentation

**FIGURE 3.**
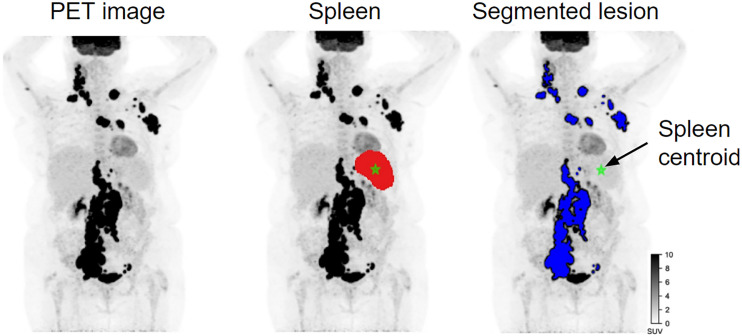
Maximum-intensity projection of PET images with spleen segmentation by AI model and lesion segmentations by expert. Cross hair indicates centroid of spleen.

Among the 295 patients available for PET/CT segmentations (regardless of the availability of survival data; Fig. 1), the TotalSegmentator AI method correctly detected and segmented the spleen in 282 patients (96%); the postprocessing method yielded a warning in 13 patients (4%) and automatically corrected the segmentation in 12 of 13 patients. The spleen segmentation of 1 patient only had to be manually corrected using the LIFEx software. The median size of the spleen was 256.15 cm^3^ (interquartile range, 169–395 cm^3^). A total of 101 (34%) patients had lesions segmented by the expert in the spleen. Figure 3 shows the coronal maximum-intensity projection views of the PET image of 1 patient, the PET image overlapped with the spleen segmentation by the AI model, and the lesion segmentations by the expert. The automatically calculated centroid of the spleen is shown as a cross hair.

### PET/CT Features

Table 2 shows the descriptive statistics of the baseline PET/CT features in 282 patients. Neither the sDspleen nor the SpreadSpleen was correlated with TMTV (ρ = 0.14 and 0.21, respectively) or IPI (ρ = 0.12 and 0.15, respectively). The correlation between sDspleen and SpreadSpleen was 0.68. All dissemination features were moderately correlated pairwise (ρ = 0.60–0.78). The correlogram between all PET/CT features is given in Supplemental Figure 2.

**TABLE 2.** Statistics of PET/CT Features

PET/CT feature	Mean	SD	Median	Interquartile range 1–3
TMTV (cm^3^)	434.43	569.52	240.02	78.24 – 551.08
SpreadSpleen (cm)	6.30	3.03	6.67	3.99–8.41
Dspleen (cm)	32.02	9.57	32.43	25.92–38.73
sDspleen × 100 (m^−1^)	17.59	5.45	18.05	13.80–21.29
Dmax (cm)	44.08	23.05	44.62	23.84–64.17
Dbulk (cm)	31.63	18.62	31.73	15.63–44.40

### Univariate Survival Analysis

The median values of Dspleen, sDspleen, and SpreadSpleen shown in Table 2 were used as cutoff values for the Kaplan–Meier survival analysis. Each feature reflecting the tumor location relative to the spleen (Dspleen, sDspleen, and SpreadSpleen) classified the patients into 2 risk groups (high risk and low risk) significantly for both PFS and OS (log‐rank test, *P* < 0.001; Supplemental Fig. 3). When the optimal cutoff values obtained by maximizing the Youden index of an AUC were used, all biomarkers were predictive of PFS and OS (log-rank test, *P* < 0.05). Of the 73 (26%) patients with PFS less than 4 y, 51 (70%) patients also had a Dspleen greater than the median value (32.43 cm), and 52 (71%) patients had a TMTV greater than the median value (242 cm^3^). The Harrell concordance indices for PFS and OS were 0.66 and 0.66 for TMTV, 0.64 and 0.60 for Dmax, 0.62 and 0.60 for Dbulk, 0.65 and 0.63 for SpreadSpleen, 0.66 and 0.64 for Dspleen, 0.65 and 0.64 for sDspleen, 0.63 and 0.63 for tumor spread measured from the liver, 0.63 and 0.63 for the distance between the liver and the farthest lesion from the liver, 0.63 and 0.63 for the standardized distance from the liver, and 0.59 and 0.61 for IPI, respectively.

The time-dependent AUC and hazard ratios with 95% CI of the image-based biomarkers are shown in Table 3. The sDspleen feature scored the highest for predicting both OS and PFS in terms of hazard ratios. All PET features and the IPI were predictive of survival in patients with DLBCL, as indicated by their AUC (≥0.60), and their predictive values were all statistically significant (*P* < 0.05).

**TABLE 3.** Univariate Analysis of the Predictive Value of PET/CT Features in Time-Dependent AUC and Hazard Ratios

	PFS		OS	
Clinical and PET/CT feature	AUC	HR	AUC	HR
Known features				
IPI	0.60 (0.54–0.66)	3.45 (1.09–8.66)	0.61 (0.53–0.69)	6.18 (1.47–17.44)
TMTV	0.67 (0.59–0.74)	12.03 (2.15–57.56)	0.67 (0.58–0.76)	18.16 (2.63–108.20)
Dmax	0.64 (0.58–0.70)	7.87 (2.04–18.87)	0.62 (0.53–0.70)	7.39 (1.43–18.38)
Dbulk	0.63 (0.55–0.69)	5.59 (1.56–14.15)	0.62 (0.53–0.70)	6.96 (1.25–18.24)
New features				
SpreadSpleen	0.64 (0.57–0.70)	5.85 (1.63–15.16)	0.63 (0.54–0.70)	9.87 (1.66–31.45)
Dspleen	0.66 (0.59–0.74)	17.49 (3.62–60.47)	0.64 (0.55–0.72)	18.18 (2.30–54.66)
sDspleen	0.66 (0.60–0.72)	53.28 (10.01–181.01)	0.65 (0.57–0.73)	32.51 (2.18–348.53)

HR = hazard ratio.

Data are mean with 95% CI.

### Multivariate Survival Analysis

**FIGURE 4.**
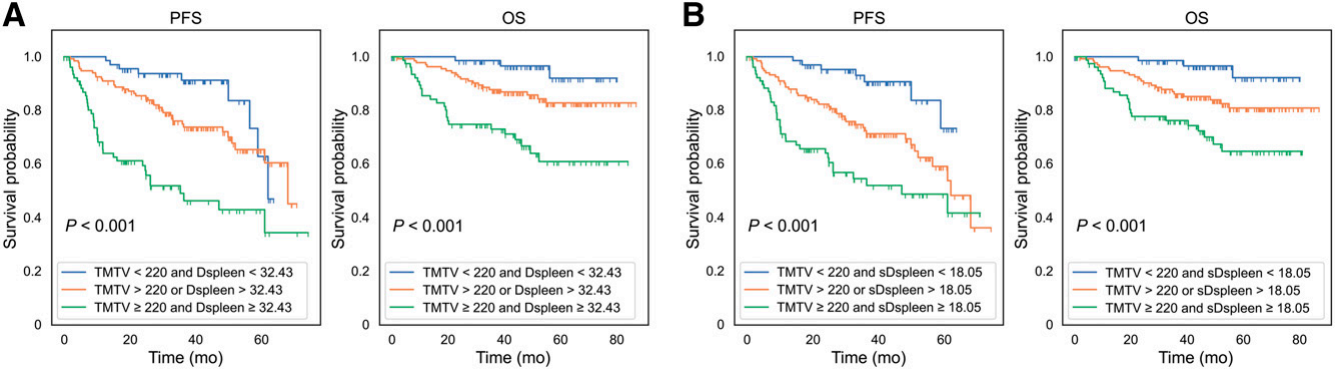
Three-risk category Kaplan–Meier curves of OS and PFS according to TMTV (cm^3^) with Dspleen (A) and sDspleen (×100 m^−1^) (B).

Three risk categories can be significantly distinguished by combining the TMTV with the Dspleen or sDspleen (Fig. 4). Supplemental Figure 4 shows the features for SpreadSpleen, Dmax, and Dbulk. For simplicity, we used the median values as the cutoff values in the Kaplan–Meier analysis for these 3 features, whereas the optimal TMTV value of 220 cm^3^ previously published was used (9). The sDspleen values were scaled by 100 for computational purposes.

Results from multivariate Cox regression analyses are shown in Table 4. We consider 3 baseline models to evaluate the added predictive value of the new PET/CT features with respect to the known biomarkers. The baseline models were based on TMTV, IPI, and TMTV combined with IPI. The added survival predictive values were evaluated using the concordance index. Only Dbulk had no significant change in the concordance index (*P* > 0.05) when combined with the TMTV for OS prediction. In all scenarios, regardless of the baseline model, the tumor location relative to the spleen (Dspleen, sDspeen, and SpreadSpleen) features significantly improved the predictive power of the IPI, TMTV, and TMTV combined with IPI (*P* < 0.05). The highest concordance indices were achieved when the spleen location-based features (SpreadSpleen, Dspleen, and sDspleen) were combined with TMTV and with the association of TMTV and IPI.

**TABLE 4.** Predictive Value of PET/CT Features, Combined with Known Features (TMTV and IPI) as Baseline Cox Model

PET/CT features	PFS (C-index)	Increase in C-index	OS (C-index)	Increase in C-index
IPI (baseline)	0.58 (0.53–0.64)		0.61 (0.53–0.68)	
IPI + TMTV	0.65 (0.58–0.71)	0.07	0.67 (0.59–0.75)	0.07
IPI + Dmax	0.65 (0.59–0.70)	0.07	0.65 (0.56–0.72)	0.04
IPI + Dbulk	0.65 (0.59–0.69)	0.06	0.66 (0.57–0.73)	0.05
IPI + SpreadSpleen	0.67(0.59–0.72)	0.08	0.68 (0.59–0.76)	0.08
IPI + Dspleen	**0.68 (0.62–0.73)**	0.09	**0.69 (0.59–0.77)**	0.08
IPI + sDspleen	0.67 (0.61–0.73)	0.09	0.68( 0.59–0.76)	0.08
TMTV (baseline)	0.66 (0.59–0.72)		0.67 (0.6–0.8)	
TMTV + Dmax	0.67 (0.58–0.72)	0.01	0.67 (0.58–0.74)	−0.01
TMTV + Dbulk	0.67 (0.59–0.72)	0.01	0.67 (0.59–0.75)	0.00
TMTV + SpleenSpread	0.69 (0.62–0.74)	0.03	0.70 (0.61–0.78)	0.03
TMTV + Dspleen	0.69 (0.62–0.74)	0.03	0.70 (0.63–0.77)	0.03
TMTV + sDspleen	**0.69 (0.62–0.75)**	0.03	**0.71 (0.64–0.78)**	0.04
TMTV + IPI (baseline)	0.65 (0.58–0.71)		0.67 (0.59–0.75)	
TMTV + IPI + Dmax	0.67 (0.60–0.72)	0.02	0.68 (0.61–0.75)	0.00
TMTV + IPI + Dbulk	0.67 (0.60–0.72)	0.02	0.68 (0.61–0.75)	0.01
TMTV + IPI + SpreadSpleen	0.69 (0.61–0.74)	0.04	0.71 (0.62–0.79)	0.03
TMTV + IPI + Dspleen	**0.69 (0.63–0.74)**	**0.04**	**0.71 (0.63–0.78)**	**0.03**
TMTV + IPI + sDspleen	0.69 (0.63–0.75)	0.04	0.71 (0.59–0.75)	0.04

C-index = concordance index.

Data are mean with 95% CI. Bold font indicates highest C-index.

Supplemental Figure 3D demonstrates that lymphoma splenic invasion is also a poor-prognosis factor, with a log-rank test showing a *P* value of 0.03 for PFS and OS. In the subgroup of patients without splenic invasion (*n* = 185), the proposed spleen-based biomarkers were prognosticators (Supplemental Fig. 5). The results in Supplemental Table 1 further confirm the improved added predictive values of the spleen-based features to the combined model of TMTV with IPI in patients without splenic invasion.

## DISCUSSION

We developed and evaluated a new framework to extract novel PET/CT features that characterize how tumors spread and disseminate relative to the spleen in patients diagnosed with DLBCL and demonstrated the additional prognostic values of these features compared with known prognostic features. Lymphoma is a cluster of blood cancers that develop from lymphocytes and can spread to various body parts through the circulatory system. The spleen, an essential organ in blood filtration, plays a crucial role in removing old or damaged red blood cells, pathogens, and other foreign particles. The spleen also acts as a reservoir for the immune cells and platelets, which are critical for fighting infections and blood clotting.

The specific role of the spleen in the lymphatic system (10) was the primary motivation for considering the spleen as a reference organ to model disease spread and dissemination. We therefore investigated the possibility of simple and interpretable features that could characterize the location of the lesions relative to the spleen in patients with DLBCL. Lesion location–based features are often less sensitive to variations in imaging modalities or imaging devices and segmentation methods once the lesions are identified, which is an asset to transfer from academic research to routine clinical practice (8).

This study introduces 3 new features (SpreadSpleen, Dspleen, and sDspleen) extracted from baseline PET/CT images to quantify tumor locations relative to the spleen. Previous studies have proven that PET/CT-based features can be used for patient management on different DLBCL cohorts (9). To the best of our knowledge, no previous studies have been conducted to model the disease distribution and dissemination relative to a reference organ. Here, we used an open-access fully automated AI algorithm to segment the spleen from the CT images (13). The segmentation results were carefully assessed, and in 96% (282/295) of the patients, the spleen segmentation was acceptable. A correction was needed for only 4% (13/295) of the patients. Our new features are based on the centroid of the spleen, which makes them barely sensitive to the precise delineation of the spleen. We checked this lack of sensitivity to the exact delineation of the spleen by randomly shifting the spleen centroid by 2 cm and observed no substantial changes in prognostic values of the spleen-based biomarkers (results provided in the supplemental material). Experts performed lesion segmentations in our study. However, to fully automate the process, the pipeline could be integrated with existing AI-based automatic lesion segmentations from PET/CT images (16–19).

Univariate and multivariate Kaplan–Meier and Cox model analyses showed that SpreadSpleen, Dspleen, and sDspleen are strong predictors of PFS and OS. They can significantly classify patients with DLBCL into 2 risk groups (high risk and low risk) using the median values as cutoff values. Experimental results showed that Dspleen predicts PFS and OS like sDspleen. In cases of missed body surface area in clinical information, Dspleen could be used. SpreadSpleen, Dspleen, and sDspleen features can be calculated for any patient, including those with a single lesion. They consistently and significantly improved the predictive power of the IPI, TMTV, and IPI combined with TMTV. The improvements were more significant than those brought by other lesion dissemination–based features (Dmax and Dbulk). It demonstrates that the spleen-based features can complement TMTV and IPI to better characterize the disease at an early stage.

The proposed spleen-based features (SpreadSpleen, Dspleen, and sDspleen) are simple to calculate and intuitive to interpret. The spleen was chosen as the reference organ because of its role in the lymphatic system. Lymphoma splenic invasion is also a poor prognosis factor. In subgroups of patients without splenic invasion, the spleen-based features were prognosticators, suggesting that the spleen-based features code more than just a patient’s splenic invasion. The bladder and liver were also tested as reference organs for evaluating tumor distribution and dissemination features. The predictive value from the bladder-based features was low for both PFS and OS (AUC < 0.60, data not shown). Although liver-based features were correlated with spleen-based features because of their relative anatomic proximity and were also predictive (data in the supplemental material), spleen-based features always had significantly higher predictive values (*P* < 0.05).

The main limitation of our study is the need for further evaluation of the proposed new features, ideally in multicenter cohorts, to confirm our findings. In addition, developing a machine-learning model that would best combine all PET/CT prognostic features to improve the prediction of PFS and OS warrants further investigation.

## CONCLUSION

In this study, we introduced 3 predictive biomarkers extracted from baseline PET/CT images in patients with DLBCL. To our knowledge, this is the first study showing that characterizing tumor location relative to a reference organ predicts survival in a large series of DLBCL patients. We demonstrated that characterizing how the tumor spreads and disseminates relative to the spleen can improve survival prediction (PFS and OS) compared with the internationally accepted risk-score method, age-adjusted IPI, and PET/CT image-based biomarker, TMTV, for patients with DLBCL.

## DISCLOSURE

The REMARC clinical studies and analyses were sponsored by the Lymphoma Academic Research Organization (LYSARC) of France. Kibrom Girum and Irène Buvat received a research grant given to the Institut Curie by ANR (ANR-19-SYME-0005-03). Louis Rebaud was employed by Siemens Medical Solutions. No other potential conflict of interest relevant to this article was reported.

## Notes

KEY POINTS **QUESTION:** Do baseline [^18^F]FDG PET/CT features that characterize the tumor location relative to a reference organ predict survival in patients with DLBCL, and can they improve the predictive value of the TMTV and age-adjusted IPI? **PERTINENT FINDINGS:** New baseline PET/CT features characterizing tumor spread and dissemination relative to the spleen predicted PFS and OS in DLBCL patients. The new biomarkers significantly enhanced the predictive value of TMTV and IPI for both PFS and OS. **IMPLICATIONS FOR PATIENT CARE:** Tumor spread and dissemination features relative to the spleen calculated from baseline [^18^F]FDG PET/CT are prognostic biomarkers in patients with DLBCL.
